# Effect of assessing velocity time integral at different locations across ventricular outflow tracts when calculating cardiac output in neonates

**DOI:** 10.1007/s00431-023-05121-x

**Published:** 2023-07-25

**Authors:** Jane Huang, Yogen Singh, Mohammad Adie, Shahab Noori, Mahmood Ebrahimi, Manuel Durand, Rowena Cayabyab, Rangasamy Ramanathan

**Affiliations:** 1 Division of Neonatology, Department of Pediatrics, LAC+USC Medical Center, Keck School of Medicine, University of Southern California, Los Angeles, CA USA; 2 Division of Neonatology, Loma Linda University School of Medicine, Loma Linda, CA USA; 3 Fetal and Neonatal Institute, Division of Neonatology, Department of Pediatrics, Keck School of Medicine, Children’s Hospital Los Angeles, University of Southern California, Los Angeles, CA USA

**Keywords:** Neonates, Echocardiography, Cardiac output, Stroke volume, Velocity time integral (VTI)

## Abstract

This study aims to evaluate the effect of assessing velocity time integral at different locations across ventricular outflow tracts for calculating cardiac output (CO) in neonates. Velocity time integral (VTI) and CO were measured at 3 different locations across right and left ventricular outflow tracts using transthoracic echocardiography in healthy term neonates without any major congenital heart disease. ANOVA with Bonferroni correction was used to determine the differences between the VTI and CO sampled at these three locations. Forty-one neonates met inclusion criteria with mean gestational age of 38.6 ± 1 weeks and mean birth weight of 3155 ± 463 g. The median hours after birth when echocardiography was obtained was 23 h (range 11–68 h after birth). Left CO were 121 ± 30 mL/kg/min, 155 ± 38 mL/kg/min, and 176 ± 36 mL/kg/min measured below the valve, hinges of the valve, and tip of the valve, respectively. Right CO were 197 ± 73 mL/kg/min, 270 ± 83 mL/kg/min, and 329 ± 104 mL/kg/min measured below the valve, hinges of the valve, and tip of the valve, respectively. A statistically significant difference (*P* < 0.001) was found in the VTI and CO measured at the 3 different locations across both left and right ventricular outflow tracts.

*     Conclusions*: There is a significant difference in measurements of VTI and CO depending on the location of Doppler gate sampling across the ventricular outflow tracts. Consistency and precision in Doppler gate location are essential for measuring VTI and calculating CO while assessing changes in hemodynamic status in critically ill infants.

**Table:** 

**What is Known:** *• Targeted Neonatal Echocardiography is increasingly applied to measure cardiac output in critically ill neonates and serial assessments are performed to assess the trend in changes in cardiac output*. *• Noninvasive measurement using velocity time integral to calculate cardiac output is commonly performed. However, location of Doppler sample gate to measure ventricular outflow tract velocity time integral is not consistent*.	
**What is New:** *• Statistically significant changes in measured velocity time integral and cardiac output are noted based on the location of Doppler gate sampling*. *• To monitor the cardiac output for trending, it is important to be consistent with regards to the location of the Doppler sample gate to assess changes in cardiac output in critically ill newborns*.

**What is Known:**

*• Targeted Neonatal Echocardiography is increasingly applied to measure cardiac output in critically ill neonates and serial assessments are performed to assess the trend in changes in cardiac output*.

*• Noninvasive measurement using velocity time integral to calculate cardiac output is commonly performed. However, location of Doppler sample gate to measure ventricular outflow tract velocity time integral is not consistent*.

**What is New:**

*• Statistically significant changes in measured velocity time integral and cardiac output are noted based on the location of Doppler gate sampling*.

*• To monitor the cardiac output for trending, it is important to be consistent with regards to the location of the Doppler sample gate to assess changes in cardiac output in critically ill newborns*.

## Introduction

In recent years, target neonatal echocardiography (TNE) has been increasingly used in neonatal intensive care units (NICUs) to evaluate the hemodynamic status in critically ill infants, and it provides a quick and non-invasive real-time assessment of cardiac function and cardiac output [1–4]. Prior studies have reported changes in clinical management in 30–60% of patients with hemodynamic instability following the use of TNE in the NICU [5].

There are several ways to measure cardiac output (CO) using echocardiography. One of the techniques commonly used is using pulsed wave Doppler to estimate stroke volume (SV) as cardiac output is the product of stroke volume and heart rate (HR). SV is estimated by obtaining ventricular outflow tract cross-sectional area (CSA) and velocity time integral (VTI) across respective left or right ventricular outflow tracts. VTI, also known as stroke distance, represents the area under the Doppler spectral curve measured across the ventricular outflow tract during one heartbeat or systole [6]. CSA is calculated by measuring the diameter (D) of the ventricular outflow tract and using the formula: $CSA=VTI\times\pi\;(D/2)^{2}$ . Cardiac output is then calculated as $CO=VTI\times\pi\;(D/2)^{2}\times HR$.

Accurate assessment of CO is thus dependent on the precise measurement of VTI and CSA as CO can vary if there is a difference in VTI obtained at various locations across the ventricular outflow tracts. The American Society of Echocardiography (ASE) in collaboration with the European Association of Echocardiography (EAE) and the Association for European Pediatric Cardiologists (AEPC) recommended Doppler sampling gate location to be just below the valve level, where cross-sectional area is measured [1]. However, there is still inconsistency in the methodology of obtaining VTI among providers or in published studies, especially when it comes to the precise location of Doppler sampling gate across the outflow tracts.

In our institution, it is a routine practice to perform TNE on all neonates admitted to newborn nursery prior to discharge with verbal consent from the parents. TNEs are performed by NICU fellows under direct supervision of a single echocardiographer with more than 30 years of experience in performing echocardiography in neonates. We noted wide variations in CO obtained with these TNE studies, which led us to conduct this quality improvement project to evaluate the effect of assessing VTI at different Doppler sampling gate locations across the ventricular outflow tracts on the CO in neonates.

## Materials and methods

All healthy term neonates born between February and May 2022 at Los Angeles County + University of Southern California (LAC + USC) Medical Center without any congenital heart disease, except for patent ductus arteriosus and patent foramen ovale, were enrolled. Maternal and neonatal demographics were obtained through electronic health record chart review. Routine TNE was performed in all eligible infants by a single experienced clinician with over 30 years of experience in performing echocardiography in neonates.

**Fig. 1:**
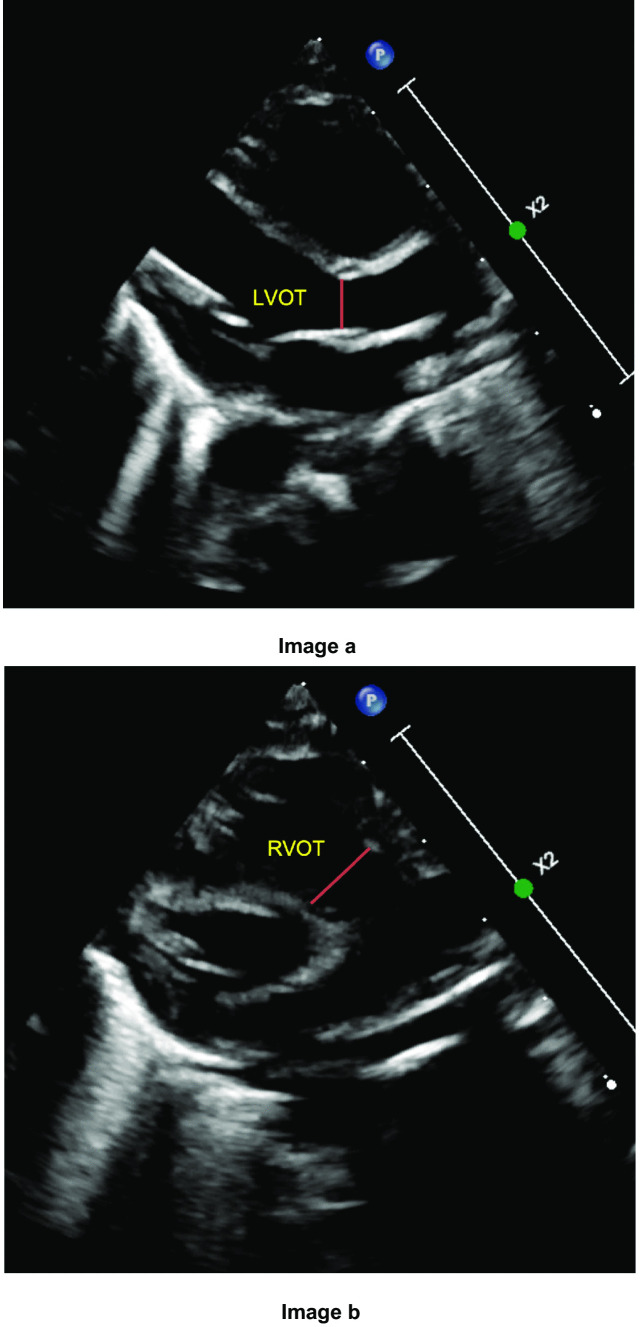
Measurement of LVOT and RVOT diameter.** a** shows LVOT in PLAX view with measurement of LVOT diameter at hinge point of AV valve (red line). **b** shows RVOT in PLAX RVOT view with measurement of RVOT diameter at hinge point of PV valve (red line). LVOT, left ventricular outflow tract. AV, aortic valve. RVOT, right ventricular outflow tract. PV, pulmonic valve. PLAX, parasternal long axis view

All echocardiography studies were performed using a Philips EPIQ model cardiovascular ultrasound machine with 9 Hz transducer. Angle correction was not used, and measurement was obtained by a single experienced clinician to minimize the interobserver variation of angle of insonation. The left and right ventricular CO were calculated by measuring the ventricular outflow tract CSA and VTI as described below. The CSA of the left or right ventricular outflow tract was determined by measuring the end-systole internal diameter of ventricular outflow tracts at the hinge point of aortic valve or pulmonic valve annulus, respectively [5]. The diameter of left ventricular outflow tract (LVOT) was measured in the parasternal long axis view (PLAX), and the diameter of right ventricular outflow tract (RVOT) was measured in the PLAX RVOT view (Fig. 1). CSA was then calculated as CSA = $(\mathrm{D}/2)^{2}\times\pi$, where D is the diameter.

**Fig. 2:**
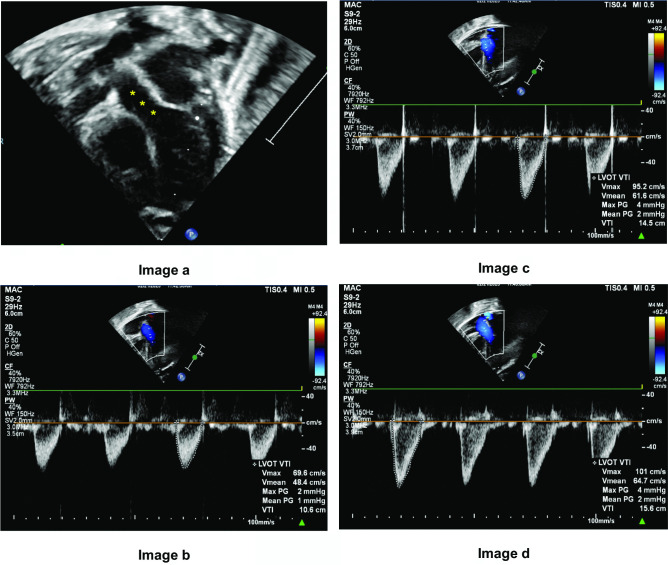
Measurement of VTI at different locations along the LVOT. **a** shows VTI measured at the 3 locations along LVOT in apical 5 chambers view (yellow asterisks). **b**–**d** show changes in VTI measurement as location of Doppler sampling gate changes. **b** shows VTI sampled below the valve. **c** shows VTI sampled at the hinges of valve. **d** shows VTI sampled at the tip of the valve. VTI, velocity time integral. LVOT, left ventricular outflow tract

**Fig. 3:**
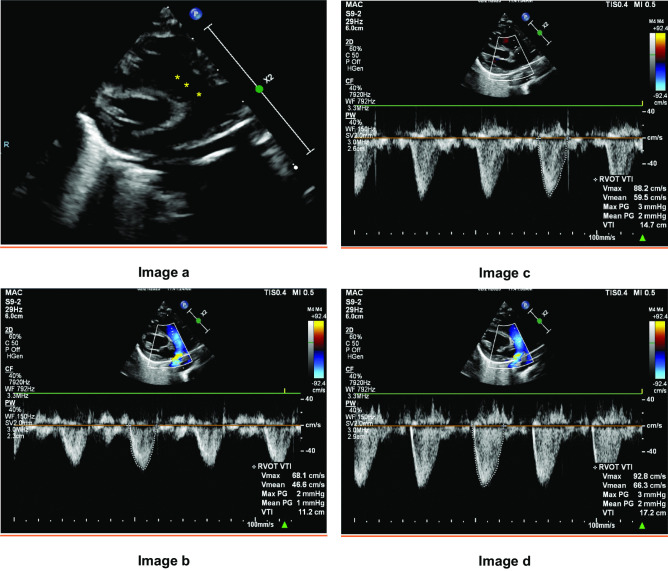
Measurement of VTI at different locations along the RVOT. **a** shows VTI measured at the 3 locations along RVOT in PLAX RVOT view (yellow asterisks). **b**–**d** show changes in VTI measurement as location of Doppler sampling gate changes. **b** shows VTI sampled below the valve. **c** shows VTI sampled at the hinges of valve. **d** shows VTI sampled at the tip of the valve. VTI, velocity time integral. RVOT, right ventricular outflow tract. PLAX, parasternal long axis view

The left ventricular VTI was measured in apical five-chambers view (Fig. 2), and the right ventricular VTI was measured in PLAX RVOT view (Fig. 3) with pulse wave Doppler spectral at the three locations across both LVOT and RVOT, 1 mm below (proximal) the valve level, at the level of valve hinges, and the tip of the aortic and pulmonic valve (distal to the valve). Heart rate (HR) was calculated automatically by the cardiovascular ultrasound machine from the ECG recording. CO can then be measured using the following formula:

$$CO\;(mL/kg/min)\hspace{0.17em}=\hspace{0.17em}\frac{VTI\;(cm) \times CSA\;({cm}^{2}) \times HR}{Weight\;(kg)}$$

All measurements were analyzed offline using EchoPAC software by a single investigator following standardization of measurement methodology discussed and agreed upon by other co-investigators. Both VTI and CSA were measured as a single measurement.

Other relevant echocardiographic data, which can affect cardiac output, was obtained including age in hours afterbirth when echocardiography was performed and presence of patent ductus arteriosus (PDA) or patent foramen ovale (PFO) and their sizes.

### Data analysis

Neonatal characteristics are presented in mean (± standard deviation, SD) and median (interquartile range, IQR) as appropriate for continuous variables and number (%) for categorical variables. Analysis of variance (ANOVA) with Bonferroni correction was used to determine the mean differences between the VTI and CO sampled at the three locations of the left and right ventricular outflow tracts. STATA 14, College Station, Texas, was used to perform data analysis. A *p*-value of < 0.05 was considered statistically significant for ANOVA. Adjusted *p*-value was < 0.017 with Bonferroni correction.

## Ethical approval

This is a prospective quality improvement project conducted at LAC + USC Medical Center approved by the Institutional Review Board at our institution with a waiver of informed consent from the parents. In our institution, it is a routine practice to obtain TNE on all neonates admitted to newborn nursery prior to discharge. Verbal consent from the parents is obtained prior to performing TNE.

## Results

Forty-one neonates met inclusion criteria during the study period, and all had TNE performed within 3 days after birth. The mean gestational age at birth was 38.6 ± 1 weeks, and the mean birth weight was 3155 ± 463 g. Demographics and characteristics of the study population are reported in Table 1. The median age in hours after birth when echocardiography was performed was 23 h (range 11–68 h). Fifty-nine percent (24/41) of neonates had PDA, and 95% (39/41) of neonates had PFO.

**Table 1:** Neonatal characteristics

**Characteristics**	***n=*41**
Gestational age (weeks)^a^	38.6 ± 1.05
Birth weight (g)^a^	3155 ± 463
Male, *n* (%)	18 (44)
Caesarian section, *n* (%)	17 (41)
Apgar score^b^ 1 min 5 min	8 (7–9) 9 (9–9)
Hour of life echocardiogram obtained^b^	23 (11–68)
PDA, *n* (%) Diameter (mm)^a^	24 (59) 0.99 ± 0.96
PFO, *n* (%) Diameter (mm)^a^	39 (95) 1.83 ± 0.81

^a^Data reported as mean ± SD

^b^Data reported as median (IQR)

 The mean ± SD of left and right ventricular outflow tract diameter, cross-sectional area, VTI, heart rate, and CO are shown in Table 2. There was a statistically significant difference between both left and right ventricular VTIs and CO when obtained at the three different locations across ventricular outflow tracts (*p* < 0.001) (Table 3). There was an average increase in left CO of 27 mL/kg/min (20% increase) and increase in right CO of 66 mL/kg/min (30% increase) every time the Doppler sampling gate moved distally across the outflow tracts from below the valve to hinges of the valve then to the tip of the valve (*p* < 0.001) (Table 3). Post hoc analysis with Bonferroni correction showed statistically significant difference between VTI and CO obtained at the three different locations (*p* < 0.017 for all) (Table 4). Mixed model for repeated measures was also performed, and the results for mean right and left CO and VTI were slightly higher after fixing for participant. However, the mean differences of the VTI and CO from the tip and below the valve compared to the hinges of the valve were similar to the results obtained with ANOVA (data not shown).

**Table 2:** Mean ± SD of the left and right ventricular outflow tract’s diameter, cross-sectional area, velocity time integral, heart rate, and cardiac output

	**Diameter**^**a**^** (mm)**	**Cross-sectional area**^**a**^** (cm**^**2**^**)**	**Velocity time integral (cm)**	**Heart rate (beats/min)**	**Cardiac output (mL/kg/min)**
**Left ventricle**	6.4 ± 0.5	0.3 ± 0.1			
Below the valve	-	-	9.1 ± 1.7	129 ± 17	121 ± 30
Hinges of the valve	-	-	11.8 ± 2.1	127 ± 18	155 ± 38
Tip of the valve	-	-	13.6 ± 1.8	125 ± 19	176 ± 36
**Right ventricle**	8.6 ± 0.9	0.6 ± 0.1			
Below the valve	-	-	7.9 ± 1.8	130 ± 17	197 ± 73
Hinges of the valve	-	-	11.1 ± 2.0	129 ± 19	270 ± 83
Tip of the valve	-	-	13.6 ± 2.2	127 ± 19	329 ± 104

Data reported as mean ± SD

^a^Diameter and cross-sectional area of the ventricular outflow tract

**Table 3:** Summary of velocity time integral and cardiac output in 41 term neonates

	**Below the valve**	**Hinges of the valve**	**Tip of the valve**	***p*-value**
**Left ventricle**				
Velocity time integral (cm)	9.1 ± 1.7	11.8 ± 2.1	13.6 ± 1.8	< 0.001
Cardiac output (mL/kg/min)	121 ± 30	155 ± 38	176 ± 36	< 0.001
**Right ventricle**				
Velocity time integral (cm)	7.9 ± 1.8	11.1 ± 2.0	13.6 ± 2.2	< 0.001
Cardiac output (mL/kg/min)	197 ± 73	270 ± 83	329 ± 104	< 0.001

Data reported as mean ± SD

One-way ANOVA *P* < 0.05

**Table 4:** Bonferroni correction for multiple comparison of mean VTI and CO

	**Group (I)**	**Group (J)**	**Mean difference (I–J)**	***p*-value**
**LVOT VTI**	Below the valve	Hinges of the valve	2.74 ± 0.4	< 0.001
	Hinges of the valve	Tip of the valve	1.87 ± 0.3	< 0.001
	Tip of the valve	Below the valve	4.60 ± 0.1	< 0.001
**LVO**	Below the valve	Hinges of the valve	34.08 ± 7.7	< 0.001
	Hinges of the valve	Tip of the valve	21.76 ± 1.6	0.016
	Tip of the valve	Below the valve	55.84 ± 6.1	< 0.001
**RVOT VTI**	Below the valve	Hinges of valve	3.15 ± 0.2	< 0.001
	Hinges of the valve	Tip of the valve	2.55 ± 0.2	< 0.001
	Tip of the valve	Below the valve	5.70 ± 0.5	< 0.001
**RVO**	Below the valve	Hinges of the valve	73.09 ± 10.7	0.001
	Hinges of the valve	Tip of the valve	59.10 ± 20.9	0.008
	Tip of the valve	Below the valve	132.19 ± 31.7	< 0.001

Data reported as mean ± SD

*VTI* velocity time integral, *CO* cardiac output, *LVOT *left ventricular outflow tract, *RVOT* right ventricular outflow tract, *LVO* left ventricular output, *RVO* right ventricular output

Bonferroni correction *P* < 0.017

## Discussion

Measurement of cardiac output is one of the most important echocardiographic parameters that helps in the evaluation of end-organ perfusion, oxygen delivery, and tissue perfusion in patients with hemodynamic instability [7]. CO is calculated by multiplying stroke volume and heart rate. Stroke volume and CO are affected by preload, afterload contractility, and the heart rate. Changes in CO from baseline often reflect changes in total body oxygen needs and metabolic demands [7]. CO often increases as a physiological response to stress to ensure adequate oxygen delivery and tissue perfusion. Low CO in the setting of hemodynamic instability may indicate inadequate systemic perfusion, and it could be from low preload, poor contractility, and/or abnormal afterload [2, 7]. Understanding the etiology of low CO is important in providing targeted timely individualized treatment. In neonates, in the absence of PDA, left ventricular CO equals systemic blood flow [5].

Several prior studies have described CO in healthy term neonates measured non-invasively via transthoracic echocardiography to be approximately 135 to 325 mL/kg/min [2, 8–10]. The reason for this variation in CO is likely related to the presence of intracardiac shunts and PDA in neonates, inconsistency in methodology in acquiring CSA and VTI of the ventricular outflow tract, and/or the timing when the echocardiography is obtained as studies have shown that CO increases over 24 h after birth and then remains stable between 24 and 72 h [11, 12]. Both Beker et al. [13] and Pereira et al. [14] described variation in left ventricular (LV) CO depending on the position where the ventricular outflow tract diameter is measured. Ihlen et al. also described variation in CO at different cross-sectional area of the ascending aorta in adults with angina pectoris and reported that the CO measurements at the aortic orifice are best correlated with that measured invasively with thermodilution and Fick method [15]. In their study, they also looked at the variation in maximum velocity obtained at the different locations along the ascending aorta and found no difference [15]. Fisher et al. reported comparable results and found no significant difference in the mean Doppler velocity measured at different locations when measured in open-chest dogs [16]. To the best of our knowledge, the degree of variations in VTI and CO measured at different locations along the ventricular outflow tracts have not been previously characterized in healthy term neonates.

Our study demonstrated that there are significant differences in the measurements of VTI and CO depending on the location of Doppler sampling gate at the ventricular outflow tracts, and this was consistent for both left and right ventricular CO and VTIs. Our results contrast with those reported by Ihlen et al. [15] and Fisher et al. [16] which may be due to differences in sampling techniques, such as the angle of insonation during Doppler assessment, echocardiographic view where VTI was obtained, or due to difference in cardiac and circulatory physiology in neonates and adults. In our study, right CO measurements were consistently higher at all 3 locations when compared to the left CO at similar locations. This finding is consistent with the published reports that higher right ventricle (RV) CO in the first 24 h after birth compared to LV CO [12, 17, 18]. One possibility for this observation is the presence of intracardiac shunt across the PFO (present in 95% of neonates) at the time of echocardiography assessment. Furthermore, the diameter and CSA of RVOT were noted to be larger compared to that of LVOT (RVOT diameter of 8.6 mm and CSA of 0.56cm^2^ versus LVOT diameter of 6.4 mm and CSA of 0.33cm^2^), contributing to a higher RV CO observed. Abushaban et al. also reported similar results where the CSA of pulmonary valve was greater than CSA of aortic valve in preterm infants less than 35 weeks of gestation [19].

We acknowledge there are several limitations with this quality improvement project. Baseline variations in VTI and CO measured in the echocardiography obtained prior to the start of this project were not evaluated for comparisons to the values measured in this study. All echocardiograms were performed by a single clinician echocardiographer with over 30 years of experience in neonatal echocardiography, which is a real strength of the study, and all echocardiography were read by a single echocardiography-trained neonatologist. However, as a result of a single operator, interobserver differences and generalizability of the results were not studied. Our sample size was relatively small and only consisted of healthy term neonates without any major congenital heart disease. We selected this cohort as our initial study population to avoid confounding variables. Most infants had contamination of left and right ventricular CO from cardiac shunts (PFO and PDA shunts). Furthermore, although this study demonstrated that there are significant variations in VTI and CO estimated non-invasively via transthoracic echocardiography depending on the sampling locations, which location best correlates with the true CO was not compared to the gold standard of invasive CO from catheter study, via direct Fick’s method, or cardiac magnetic resonance imaging. However, there are several studies in both neonates and adults that showed CO measurements obtained via transthoracic echocardiography are consistent with invasively obtained measurements [6, 10, 20–22].

In conclusion, there are significant differences in both left and right ventricle VTIs and CO measured at different locations along the respective ventricular outflow tracts. Therefore, consistency and precision in the Doppler sampling gate location is critical for accurately measuring VTI and CO while assessing changes in hemodynamic status; as changes in CO may be related to the location of Doppler gate sampling rather than changes in hemodynamic status in critically ill newborns. This becomes even more important when intervention is based upon trending of VTI and CO measurements. Following this project, we will establish and implement unit protocol, so consistent methodology is used in obtaining VTI and CO with transthoracic echocardiography to ensure clinical decisions are based on accurate measurement of CO. We recommend performing a larger study to confirm our findings and test these results in the preterm infants and those with hemodynamic instability.

## Abbreviations

ANOVA: Analysis of variance
AV: Aortic valve
CO: Cardiac output
CSA: Cross-sectional area
D: Diameter
HR: Heart rate
IQR: Interquartile range
LV: Left ventricle
LVOT: Left ventricular outflow tract
NICU: Neonatal intensive care unit
PLAX: Parasternal long axis view
PDA: Patent ductus arteriosus
PFO: Patent foramen ovale
PV: Pulmonic valve
RV: Right ventricle
RVOT: Right ventricular outflow tract
SD: Standard deviation
SV: Stroke volume
TNE: Target neonatal echocardiography
VTI: Velocity time integral
